# Columbianadin Suppresses Lipopolysaccharide (LPS)-Induced Inflammation and Apoptosis through the *NOD1* Pathway

**DOI:** 10.3390/molecules24030549

**Published:** 2019-02-02

**Authors:** Chao Zhang, Alan Chen-Yu Hsu, He Pan, Yinuo Gu, Xu Zuo, Bing Dong, Ziyan Wang, Jingtong Zheng, Junying Lu, Ruipeng Zheng, Fang Wang

**Affiliations:** 1 Department of Pathogeny Biology, College of Basic Medical Sciences, Jilin University, Changchun 130021, China; chaoz15@mails.jlu.edu.cn (C.Z.); panhe18@mails.jlu.edu.cn (H.P.); guyn18@mails.jlu.edu.cn (Y.G.); zuoxu18@mails.jlu.edu.cn (X.Z.); dongbing18@mails.jlu.edu.cn (B.D.); wzy16@mails.jlu.edu.cn (Z.W.); wszjt1@163.com (J.Z.); jylu15@mails.jlu.edu.cn (J.L.); 27040314@163.com (R.Z.); 2 Priority Research Centre for Asthma and Respiratory Diseases, Hunter Medical Research Institute and University of Newcastle, Newcastle 2308, Australia; alan.hsu@newcastle.edu.au

**Keywords:** columbianadin, inflammation, NOD1, NF-κB

## Abstract

Columbianadin (CBN) is one of the main bioactive constituents isolated from the root of *Angelica pubescens.* Although the anti-inflammatory activity of CBN has been reported, the underpinning mechanism of this remains unclear. In this study, we investigated the anti-inflammatory effect of CBN on lipopolysaccharide (LPS)-stimulated THP-1 cells and explored the possible underlying molecular mechanisms. The results showed that CBN suppressed LPS-mediated inflammatory response mainly through the inactivation of the NOD1 and NF-κB p65 signaling pathways. Knockdown of NOD1 reduced the degree to which inflammatory cytokines decreased following CBN treatment, whereas forced expression of NOD1 and CBN treatment reduced NF-κB p65 activation and the secretion of inflammatory cytokines. Furthermore, CBN significantly reduced cellular apoptosis by inhibiting the NOD1 pathway. Collectively, our results indicate that CBN suppressed the LPS-mediated inflammatory response by inhibiting NOD1/NF-κB activation. Further investigations are required to determine the mechanisms of action of CBN in the inhibition of NOD signaling: However, CBN may be employed as a therapeutic agent for multiple inflammatory diseases.

## 1. Introduction

Columbianadin (CBN) is a natural coumarin compound isolated from the root of *Angelica pubescens* [1]. *A. pubescens* is widely distributed in the south of China, and is the dry root of *A. pubescens* Maxim. f. biserrata Shan et Yuan. It is mainly used to treat rheumatism spasm [2] and headache in clinics, according to *Chinese Pharmacopoeia* [v2015] [3]. The major active ingredients isolated from this plant are various coumarins, including angelol, umbelliferone, CBN, angelol-B, and isoanglol [4,5]. CBN is one of the main components among the bioconstituents of *A. pubescens*. CBN has been reported to reduce inflammation [6]. Previous studies have shown that CBN inhibited the edema induced by carrageenan and the increase in vascular permeability induced by 0.25% acetic acid in mice. Another study has also reported that CBN reduced inflammation in a lipopolysaccharide (LPS)-induced lung inflammation mouse model [7]. Nevertheless, the underlying mechanisms of the anti-inflammatory effect of CBN are unclear.

Inflammation is a complex protective mechanism in response to pathogenic microorganisms and is orchestrated by epithelial and inflammatory cells through series of complicated signaling pathways. Pattern recognition receptors (PRRs), such as nucleotide-binding oligomerization domain (NOD)-like receptors (NLRs) and toll-like receptors (TLRs), are involved in the activation of inflammatory responses [8,9,10]. Intracellular NLR family members such as NLRP1, NLRP3, NLRP6, and NLRC4 mediate the assembly of inflammasome complexes that result in the activation of pro-caspase-1, which is required for the activation and release of the active and potent inflammatory cytokine interleukin (IL)-1*β* [11]. NOD1 is another member of the NOD family, and is a PRR expressed in cells of both haematopoietic and non-haematopoietic origin, including endothelial cells, where it has been shown to be critical in pathogen recognition [12]. After microbial infection, NOD1 interacts with the adaptor protein receptor-interacting protein 2 (RIP2), facilitating the formation of a multiprotein signaling platform known as the inflammasome, which activates NF-κB and facilitates the production of pro-inflammatory expression such as IL-1*β*, IL-6, and TNF-*α*. This is also known as the NOD1-RIP2-NF-κB inflammation signal [13]. LPS, a major component of the gram-negative bacterial cell wall, is a known inducer of inflammation in vitro and in vivo via TLR4-MyD88, and NOD1-RIP2-NF-κB signaling [14] could induce the secretion of inflammatory cytokines [15]. 

Mononuclear cells are intrinsic immune cells that play important roles in innate immune responses after being stimulated by LPS during microbial infection, such as antigen presentation, phagocytosis, and secretion of inflammatory mediators [16]. A previous study has shown that CBN inhibited the production of pro-inflammatory cytokines in the substance P-stimulated human mast cell line HMC-1 and reduced inflammation in mast cell-mediated allergic inflammatory responses [17].

In this study, we investigated the effects of CBN isolated from *A. pubescens* in LPS-induced inflammation in THP-1 cells. We used targeted PCR arrays and showed that CBN reduced LPS-mediated production of pro-inflammatory cytokines via the NOD1 signaling pathway. NOD1 knockdown or forced expression confirmed that CBN-mediated suppression of NF-κB p65 activation and inflammatory cytokine production was dependent on NOD1. This indicated that the anti-inflammatory effect of CBN was dependent on NOD1 and may be employed as an alternative anti-inflammatory therapeutic in chronic inflammatory diseases such as asthma and chronic obstructive pulmonary disease.

## 2. Results

### 2.1. CBN Reduced the Expression of Inflammatory Cytokines Induced by LPS in THP-1

**Figure 1:**
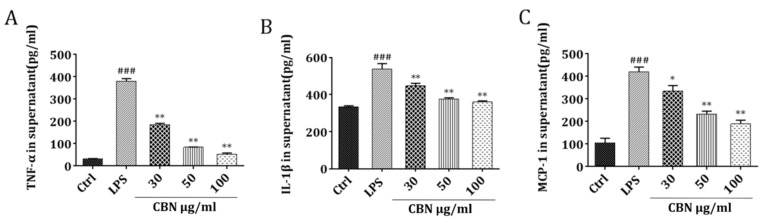
The effect of columbianadin (CBN) on the production of TNF-*α*, IL-1*β*, and MCP-1 by lipopolysaccharide (LPS) stimulation. THP-1 cells were treated with CBN for 24 h post-LPS stimulation: (**A**) TNF-*α*, (**B**) IL-1*β*, (**C**) MCP-1 levels. ### *P* < 0.001 versus control group; * *P* < 0.05 versus LPS group; ** *P* < 0.01 versus LPS group. The results shown are representative of three independent experiments with similar results. Data are expressed as means ± SD.

LPS stimulation significantly increased the protein production of pro-inflammatory cytokines TNF-*α*, IL-1*β*, and MCP-1 compared to the control group (*P* < 0.001). CBN treatment significantly inhibited LPS-stimulated inflammatory cytokine expression in a dose-dependent manner (30 μg/mL (*P* < 0.05), 50 μg/mL (*P* < 0.01), and 100 μg/mL (*P* < 0.01); Figure 1A–C). The differences in the regulating effects on the inflammatory cytokines between 50 μg/mL and 100 μg/mL CBN were not remarkable. Choosing a smaller concentration is helpful in reducing the effect of a drug on cell status. Thus, 50 μg/mL was selected for all subsequent experiments.

### 2.2. CBN Targeted the NOD1 Signaling Pathway in LPS-Induced Inflammation 

**Figure 2:**
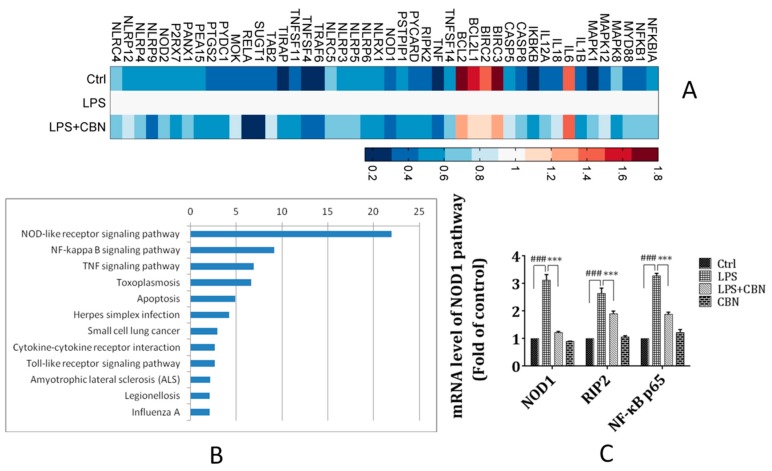
A PCR array detected that NOD1 was the main pathway affected by CBN in LPS-mediated inflammation. (**A**) The cluster analysis showed 84 inflammation-related genes were enriched after CBN treatment. Different colors indicate the fold change compared to *β*-actin (fold change > 1.5). Heatmap color intensity depicts the multiple of gene up- (41 genes) and downregulation (5 genes). (**B**) Pathway analysis of differentially expressed genes in the Kyoto Encyclopedia of Genes and Genomes (KEGG) database. Differentially expressed genes were mainly concentrated in nucleotide-binding oligomerization domain (NOD)-like receptor signaling pathways and demonstrated the highest enrichment score. The bar plot “-Log 10 (*P*-value)” refers to the enrichment score of the significant enrichment pathways. (**C**) CBN inhibited LPS-stimulated *NOD1/RIP2/NF-κB p65* pathway gene expression. ### *P* < 0.001 versus control group; *** *P* < 0.001 versus LPS group. The results shown are representative of three independent experiments with similar results. Data are expressed as means ± SD.

To investigate the signaling pathways that were affected by CBN, the CBN-treated LPS-stimulated THP-1 cells were subjected to the inflammatory PCR arrays. MatLab analysis showed 46 genes that were differentially expressed by LPS stimulation (approximately 54.76%, fold change > 1.5) (Figure 2A). CBN treatment significantly downregulated 41 genes that were upregulated by LPS and upregulated 5 genes that were downregulated by LPS.

To elucidate the pathways involved in the anti-inflammatory activity of CBN, enrichment analysis for the differentially expressed genes was performed against the Kyoto Encyclopedia of Genes and Genomes (KEGG) pathway database. This analysis showed that these differentially expressed genes were enriched in 12 different signaling pathways, among which the NOD-like receptor signaling pathway and downstream *NF-κB p65* activation were highly enriched (Figure 2B).

We then confirmed the expression of the genes involved in the *NOD1* and *NF-κB p65* signaling pathways. LPS stimulation significantly increased the gene expression of *NOD1*, *RIP2*, and *NF-κB p65*, which were markedly reduced by CBN treatment (Figure 2C). Moreover, after the treatment with CBN, the expression of *NOD1*, *RIP2*, and *NF-κB p65* was remarkably declined.

### 2.3. CBN Failed to Inhibit the Inflammatory Response in NOD1 Knockdown

**Figure 3:**
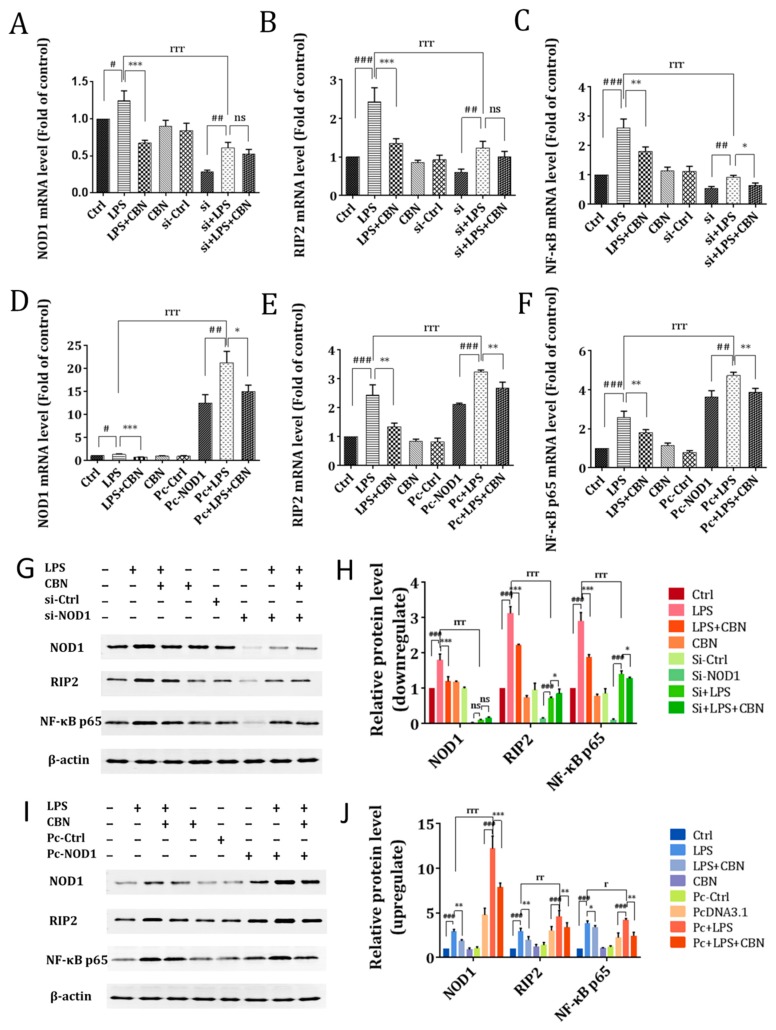
CBN inhibited the gene and protein expressions of the *NOD1/RIP2/NF-κB* pathways by blocking NOD1 activation. (**A**–**F**) indicate the gene expressions of the *NOD1* pathway. (**A**) *NOD1*, (**B**) *RIP2*, and (**C**) *NF-κB p65* gene expressions after NOD1 siRNA transfection in THP-1 cells. (**D**) *NOD1*, (**E**) *RIP2*, and (**F**) NF-κB p65 gene expressions after pcDNA-*NOD1* transfection in THP-1 cells. (**G**–**J**) indicate the protein level and relative expression of the *NOD1* pathway after *NOD1* knockout (**G**,**H**) and forced expression of *NOD1* (**I**,**J**). # *P* < 0.05, LPS group versus control group; ## *P* < 0.01, LPS group versus control group; ### *P* < 0.001, LPS group versus control group; * *P* < 0.05, drug group versus LPS group; ** *P* < 0.01, drug group versus LPS group; *** *P* < 0.001, drug group versus LPS group; r *P* < 0.05, (si + LPS/Pc + LPS)(si: *NOD1* gene knockout; Pc: *NOD1* gene overexpression) versus LPS group; rr *P* < 0.01, (si + LPS/Pc + LPS) versus LPS group; rrr *P* < 0.001 (si + LPS/Pc + LPS) versus LPS group; ns indicates no significance between the two groups. The results shown are representative of three independent experiments with similar results. Data are expressed as means ± SD.

Our results indicated that the *NOD1* pathway was involved in the anti-inflammatory activity of CBN. To further confirm the role of the *NOD1* pathway in CBN-mediated inhibition of inflammatory response, we transfected specific NOD1 siRNA or pcDNA-NOD1 expression plasmid into THP-1 cells before LPS stimulation and CBN treatment. Here, qPCR and immunoblot analysis indicated that the RNA and protein levels of *NOD1*, *RIP2*, and *NF-κB p65* in the LPS-stimulated and CBN-treated (LPS + CBN) group were significantly downregulated compared to the LPS group. When NOD1 was silenced, *NOD1*, *RIP2*, and *NF-κB p65* gene and protein expression were significantly reduced following LPS stimulation (si + LPS vs. LPS) (Figure 3A–C,G–H) (si: *NOD1* gene knockout). However, CBN treatment further reduced NF-κB p65, and not NOD1 and RIP2 expression, in LPS-treated, *NOD1*-silenced THP1 cells (si + LPS + CBN vs. si + LPS). In contrast, NOD1 ectopic expression followed by LPS stimulation led to a significant increase in RIP2 and NF-κB p65 gene expression (Pc + LPS vs. LPS) (Pc: *NOD1* gene overexpression), which was reduced by CBN treatment (Pc + LPS + CBN vs. Pc + LPS) (Figure 3D–F,I–J). These results indicated that *NOD1* was important in the *NF-κB p65* pathway, and CBN could target both *NOD1* and *NF-κB p65* expression.

### 2.4. CBN Inhibited the Expression of Pro-Inflammatory Cytokine Production

**Figure 4:**
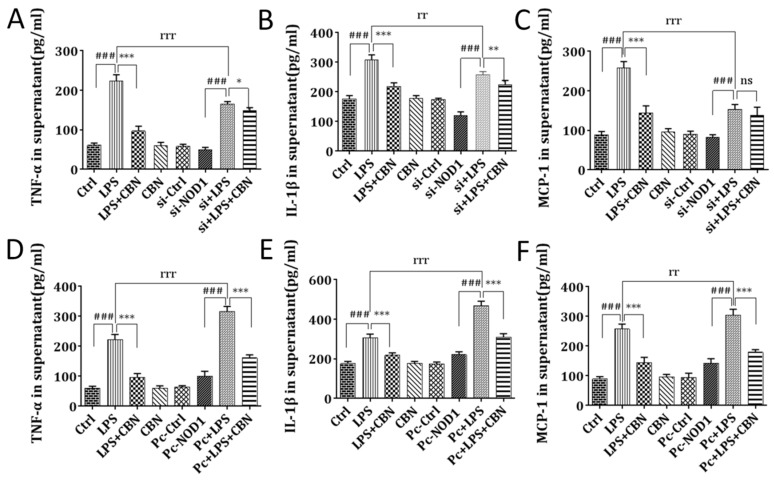
Effects of CBN on inflammatory cytokine expression in the supernatants of LPS-stimulated THP-1 cells. (**A**–**C**) Protein expression after NOD1 siRNA transfection in THP-1 cells: (**A**) TNF-*α*, (**B**) IL-1*β*, and (**C**) MCP-1. (**D**–**F**) Protein expression after pcDNA-NOD1 transfection in THP-1 cells: (**D**) TNF-*α*, (**E**) IL-1*β*, and (**F**) MCP-1. ### *P* < 0.001, LPS group versus control group; * *P* < 0.05, drug group versus LPS group; ** *P* < 0.01, drug group versus LPS group; *** *P* < 0.001, drug group versus LPS group; rr *P* < 0.01 (si + LPS/Pc + LPS) versus LPS group; rrr *P* < 0.001 (si + LPS/Pc + LPS) versus LPS group; ns indicates no significance between the two groups. The results shown are representative of three independent experiments with similar results. Data are expressed as means ± SD.

To determine if CBN-mediated inhibition of *RIP2* and *NF-κB* led to reduced production of pro-inflammatory cytokines, we also measured the levels of TNF-*α*, IL-1*β*, and MCP-1 in LPS- and CBN-treated cells. Consistently, CBN treatment significantly reduced LPS-mediated inductions of TNF-*α*, IL-1*β*, and MCP-1 (LPS + CBN vs. LPS) (Figure 4). *NOD1* knockdown significantly reduced the expression of these pro-inflammatory cytokines induced by LPS stimulation compared to *NOD1*-intact LPS-stimulated cells (si + LPS vs. LPS) (Figure 4A–C). However, there were only modest decreases in the levels of these cytokines by CBN treatment in LPS-stimulated NOD1-silenced cells compared to non-CBN-treated, LPS-treated, NOD1-silenced cells (si + LPS + CBN vs. si + LPS) (Figure 4A–C). Ectopic expression of NOD1 followed by LPS stimulation resulted in a significant increase in the levels of these cytokines, which were dramatically inhibited by CBN treatment (Pc + LPS + CBN vs. Pc + LPS) (Figure 4D,F). This indicated that the anti-inflammatory effects of CBN were dependent on *NOD1*. 

### 2.5. CBN Suppressed LPS-Induced Apoptosis via Inhibiting the NOD1 Pathway

**Figure 5:**
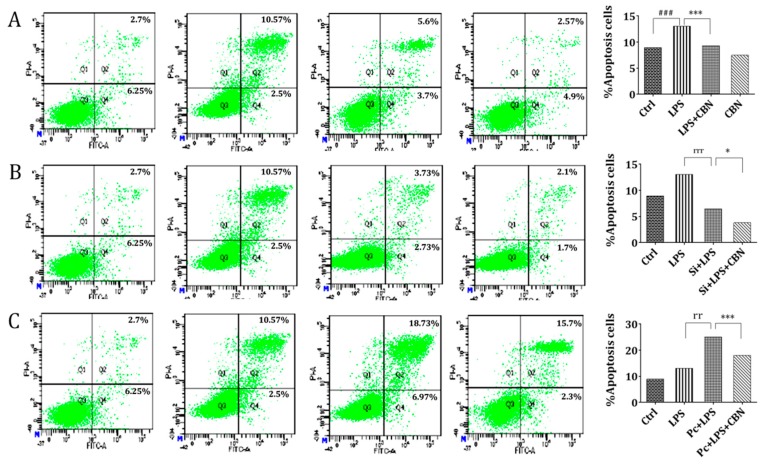
Effect of CBN on apoptosis in LPS-induced THP-1 cells. Cells were stimulated by LPS for 24 h, then incubated with indicated doses of CBN for 24 h. Annexin V/PI staining was used for apoptotic cells. Q1 represents dead cells, Q2 represents nonviable apoptotic cells, Q3 represents normal cells, and Q4 represents viable apoptotic cells. (**A**) CBN restrained LPS-induced THP-1 cell apoptosis. (**B**) The inhibiting effect of CBN on apoptosis was weakened after NOD1 siRNA transfection in THP-1 cells. (**C**) CBN showed a remarkable restrictive effect on apoptosis again after pcDNA-NOD1 transfection in THP-1 cells. ### *P* < 0.001 LPS group versus control group; * *P* < 0.05 drug group versus LPS group; *** *P* < 0.001 drug group versus LPS group; rr *P* < 0.01 Pc + LPS versus LPS group; rrr *P* < 0.001 si + LPS versus LPS group. The results shown are representative of three independent experiments with similar results. Data are expressed as means ± SD.

To evaluate whether NOD1 and CBN modulate apoptosis, we assessed the levels of apoptosis induced by LPS and CBN in THP-1 cells. LPS stimulation resulted in a significant increase (four-fold) in the apoptosis levels (LPS vs. Ctrl), which was reduced following CBN treatment (LPS + CBN vs. LPS) (Figure 5A). CBN alone did not affect cell viability and apoptosis. 

In NOD1-silenced cells, CBN treatment significantly reduced the level of apoptosis, although to a lesser extent (0.59-fold) compared to the non-CBN-treated, LPS-treated group (si + LPS + CBN vs. si + LPS) (Figure 5B). In contrast, when *NOD1* was ectopically expressed, CBN treatment reduced the level of apoptosis by approximately 7.7% compared to the non-CBN-treated, LPS-treated group (Pc + LPS + CBN vs. Pc + LPS) (Figure 5C). These data indicated that *NOD1* was one of the key targets of CBN in controlling apoptosis in LPS stimulation in THP-1 cells. 

## 3. Discussion

This study showed that CBN possessed an anti-inflammatory effect on LPS-induced inflammation in a human monocytic cell line, and we demonstrated for the first time the mechanism of the anti-inflammatory effects of CBN. We showed that the *NOD1* signaling pathway was important in the *NF-κB*-mediated induction of pro-inflammatory cytokines, and CBN treatment was a potent inhibitor of this inflammatory pathway. We also showed that *NOD1* was an important regulator of apoptosis and that CBN treatment also suppressed LPS-mediated apoptosis. 

When pathogenic microbial infections or tissue damage occur, the innate immune system is activated by the host PRRs through recognition of foreign moieties, resulting in the release of pro-inflammatory cytokines such as TNF-*α*, IL-1*β*, and MCP-1. TNF-*α* and IL-1*β* are both Th1 cytokines, which can stimulate immune cells and promote a pro-inflammatory response [18,19]. MCP-1 is a chemokine that mainly recruits monocytes, macrophages, lymphocytes, and natural killer (NK) cells to the inflamed site. MCP-1 also promotes migration, proliferation, and differentiation of leukocytes [20]. All of these immune cells and inflammatory cytokines synergistically contribute to the formation and maintenance of the inflammatory microenvironment during pathogenic microbial infections. Macrophages secrete high levels of these pro-inflammatory cytokines upon exposure to pathogens.

**Figure 6:**
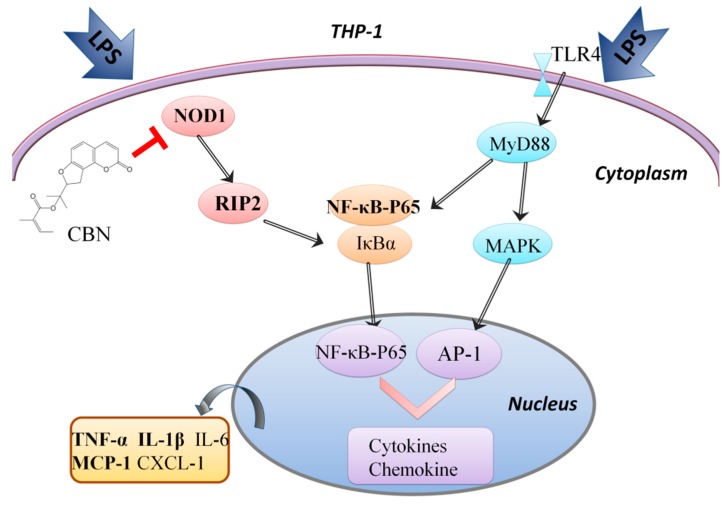
The NOD1 and TLR4 signaling pathways and the main target of CBN in the suppression of LPS-induced inflammation.

NLR is an important PRR that detects intracellular pathogens and is involved in the antibacterial response [21]. NOD1 and NOD2 are the two main members of the NLR family and are ubiquitously expressed. During bacterial infection, NOD1 recognizes foreign bacterial DNA and activates NF-κB via the signaling adaptor protein RIP2 and IKK complexes, thereby inducing the production of pro-inflammatory cytokines. NOD1 can also be activated by TNF-*α*, the production of which is dependent on TLR4 signaling. TLR4 is a PRR on the cell surface that recognizes and responds to bacterial LPS [22]. Once bound to foreign ligands, TLR4 also activates NF-κB and activator protein-1 (AP-1) to induce the production of inflammatory mediators such as IL-12, IL-18, and IL-23. Likewise, qPCR showed that the expression of TLR4 was significantly increased by LPS as NOD1 (shown in Supplementary Materials), and while both were reduced by CBN, the level of reduction of NOD1 was more pronounced than TLR4. It is possible that CBN primarily exerts its anti-inflammatory effects on the NOD1 signaling pathway, with the TLR4 pathway being a secondary target (Figure 6).

In 1995, Chen et al. [6] isolated and identified 16 compounds from the roots of *A. pubescens* and demonstrated that CBN had significant anti-inflammatory effects and analgesic activities at 10 mg/kg. The anti-inflammatory effect of CBN was also reported by Lim et al. in 2014 [7]. They investigated the anti-inflammation effect of five coumarin derivatives isolated from the roots of *A. pubescens* and demonstrated that CBN reduced the expression of nitric oxide (NO) and ameliorated the production of the inflammatory response in an in vitro model. Consistently, we also showed that CBN had a significant anti-inflammatory effect.

NOD1 signaling participated in the modulation of inflammatory processes. CBN treatment in NOD1-silenced cells showed limited inhibition of cytokine production induced by LPS. This indicates that CBN exerted its anti-inflammatory effects on the NOD1 pathway. NOD1 has also been shown to be involved in apoptosis [23]. We found that CBN decreased LPS-induced apoptosis. This reduction in apoptosis was lost when NOD1 expression was silenced. Therefore, we hold the opinion that the anti-apoptosis effect of CBN may be related to the NOD1 pathway, but we cannot say that CBN showed an anti-apoptosis effect through NOD1 gene, maybe some downstream genes played a role. At present, there are two possible explanations for this phenomenon. The first is about the inflammation. Perhaps CBN acted as an anti-inflammatory, and apoptosis was relieved because of the reduced level of inflammation. Another possibility is that NOD1 was not the critical factor to activate apoptosis here, but it significantly enhanced apoptosis induced by the activation of caspase-9 or NF-κB [24,25]. What directly affected apoptosis were the downstream factors activated by NOD1.

PSMA7 [26] has recently been shown to reduce NOD1 activation and inhibit apoptosis induction. The authors hold the opinion that there are extensive cross-talks between apoptosis and the NF-κB pathway. The existing experiment was not enough to explain the mechanism of this phenomenon: We will investigate this in-depth and comprehensively in future studies.

Collectively, our study demonstrated for the first time that CBN, an *A. pubescens* root extract, processed an anti-inflammatory and anti-apoptotic effect in human monocytic THP-1 cells. CBN suppressed both inflammatory and apoptotic response by targeting NOD1 expression upon exposure to LPS. The results presented in this study are significant, as CBN may be used as a novel and effective anti-inflammatory agent, and further studies in its clinical application will be important for chronic inflammatory diseases such as asthma.

## 4. Method

### 4.1. Cell Culture and Treatment

THP-1 cells were obtained from the cell bank of the Chinese Academy of Science (Shanghai, China) and were adjusted to 1 × 10^5^ cells/well in Roswell Park Memorial Institute (RPMI) medium modified (HyClone, Logan, UT, USA) with fetal bovine serum (GIBCO, Invitrogen, Waltham, MA, USA) and *β*-Mer 0.05 mM (Invitrogen, CA, USA). LPS (From *Escherichia coli* O55:B5) was used to treat cells at 0.1 μg/mL (Sigma-Aldrich, MO, St. Louis, state abbreviation, USA) for 24 h to induce inflammation. CBN (C19H20O5) was obtained from Solarbio (CAS:5058-13-9, Beijing, China). Cells were treated with CBN (at 30 µg/mL, 50 µg/mL, and 100 µg/mL) for 24 h after the stimulation of LPS. 

### 4.2. siRNA and Expression Plasmid Transfection

Small interfering RNA (siRNA; 5′-GGUUUAUACAACAACCAGAtt-3′) oligonucleotides for NOD1 and control siRNA (GenePharma, Shanghai, China) were transfected into THP-1 cells using Lipofectamine^TM^ RNAiMAX (Invitrogen, CA, USA) according to the manufacturer’s instructions. In addition, pcDNA-NOD1 expression plasmids (ThermoFisher, Waltham, MA, USA) and a pcDNA control vector were transfected into THP-1 cells using a Lipofectamine^TM^ 3000.

### 4.3. PCR Array

Gene expression analysis of 84 inflammation-related genes was performed using quantitative real-time PCR (qPCR) arrays based on the manufacturer’s instructions. Total RNA was isolated using an RNeasy Mini Kit, and the concentration of the extracted RNA was then quantified by measuring the absorbance at 260 and 280 nm. In addition, cDNA synthesis was performed by a Reverse Transcription Kit of 20 ng total RNA, then combined with SYBR Green Master Mix (Qiagen, New York, NY, USA) in 96-well plates following the manufacturer’s instructions. A Human Inflammasomes PCR Array, an RNeasy Mini Kit, an RNase-free DNase Set, a Reverse Transcription Kit, and SYBR Premix Ex Taq II were all obtained from Qiagen (New York, NY, USA). Thermal cycling was performed using an ABI Prism SDS 7300 system (Applied Biosystems, Waltham, MA, USA).

### 4.4. Enrichment and Pathway Analysis of Differentially Expressed Genes

Gene expression was analyzed with the ∆∆CT (Cycle threshold) method. Screening of differentially expressed genes was based on a standard fold change (FC) ≥ 2, or a fold change ≤ 0.5. To investigate the pathways of CBN-targeted inflammatory genes in LPS-stimulated THP-1, enrichment analysis of KEGG pathways was conducted in DAVID 6.8 (LHRI, Frederick, MD, USA) [27]. The significance of the enrichment was determined by *P*-values. The pathways were screened in the differentially expressed genes using enrichment scores (−Log10 (*P*-value)). Pathways were considered significant when the statistical significance reached *P* < 0.05.

### 4.5. qPCR Analysis

Total RNA was extracted by the RNeasy Mini Kit. RNA samples were reversely transcribed into cDNA by a PrimeScript^TM^ RT reagent Kit with a gDNA Eraser (Qiagen, New York, NY, USA). GAPDH (*β*-actin) expression served as the internal control. Here, qPCR was performed using SYBR Premix Ex Taq II and an ABI Prism SDS 7300 system. The reaction conditions were set as follows: 95 °C (10 min), 95 °C (1 min), 55 °C (30 s), and 72 °C (45 s) (39 cycles).

### 4.6. Immunoblot 

Intracellular proteins were extracted from THP-1 cells using ice-cold RIPA Lysis Buffer supplemented with a protease inhibitor cocktail (Roche, Basel, Switzerland). The protein concentration was detected by a bicinchoninic acid (BCA) kit. RIPA Lyses Buffer and BCA were obtained from the Beyotime Institute of Biotechnology (Shanghai, China). Proteins in the cell lysates were separated by sodium dodecyl sulfate polyacrylamide gel electrophoresis (SDS-PAGE) and then transferred to polyvinylidene fluoride (PVDF) membranes (GE Healthcare, Chicago, IL, USA). NOD1, RIP-2, NF-κB p65, and *β*-actin proteins were detected using anti-NOD1 (abcam, ab105338), RIP-2 (abcam, ab155529), NF-κB p65 (abcam, ab16502), and *β*-actin antibodies (Bioss, Cat # bs-0061R). The membranes were imaged using an enhanced chemiluminescence reagent (Merck KGaA, Darmstadt, Germany) by an imaging apparatus (AI 600 RGB, USA), according to the manufacturers’ protocol. 

### 4.7. ELISA Analysis

Cytokine levels of TNF-*α*, IL-1*β*, and MCP-1 in the culture supernatants above were assayed by an ELISA kit (RayBiotech, Guangzhou, China) according to the manufacturer’s protocol. 

### 4.8. Apoptosis

An apoptosis assay was performed after 48 h transfection with pcDNA-NOD1 plasmid or NOD1 siRNA into THP-1 cells using an Annexin V FITC/PI Apoptosis Detection Kit (BD Biosciences, Franklin Lakes, NJ, USA), and was then evaluated with an Accuri C6 flow cytometer (BD Biosciences, NJ, USA) and Cell Quest Pro Software (BD Biosciences, NJ, USA). Annexin V was combined with phosphatidylserine (PS) in the cell membrane of a viable apoptotic cell, we marked Annexin V with fluorescein isothiocyanate (FITC), which was used as a fluorescence probe. The nuclei of nonviable apoptotic cells and dead cells were marked by propidium Iodide (PI). Combined, both Annexin V and PI can be used to distinguish early apoptotic cells and late apoptotic cells. In this study, we analyzed the sum of early apoptosis and late apoptosis.

### 4.9. Statistical Analysis

All statistical analyses were expressed as mean ± SD. *T*-tests were used to compare differences between two groups. One-way analysis of variance (ANOVA) or a Mann–Whitney *U* test were used to compare differences in cytokines and mRNA using GraphPad Prism 6.02 software (Inc. La Jolla, San Diego, CA, USA). *p* **<  0.05 was considered to be statistically significant.

## Supplementary Materials

The Supplementary Materials are available online.
