# Supplementary material for: Columbianadin Suppresses Lipopolysaccharide (LPS)-Induced Inflammation and Apoptosis through the NOD1 Pathway
Source: Molecules. 2019 Feb 2;24(3):549. doi: 10.3390/molecules24030549 (PMC6384818; doi:10.3390/molecules24030549)
Supplement: Supplementary file 1 [file molecules-24-00549-s001.zip › 1 Supplementary Files TLR4.pdf]

The effect of CBN on the expression levels of TLR4

Method：

qPCR Analysis Total RNA was extracted by the RNeasy Mini Kit. RNA samples were reversely transcribed into cDNA by PrimeScript ™RT reagent Kit with gDNA Eraser (Qiagen, NYC, USA). GAPDH expression served as the internal control. qPCR was performed using the SYBR Premix Ex Taq II and an ABI Prism SDS 7300 system. The reaction conditions were set as follows: 95°C, 10 min, 95°C, 1 min, 55°C, 30 s, and 72°C, 45 s (39 cycles).

Primer sequence： TLR4 Forward 5’- TGGATACGTTTCCTTATAAG-3’

Reverse 5’-GAAATGGAGGCACCCCTTC-3’

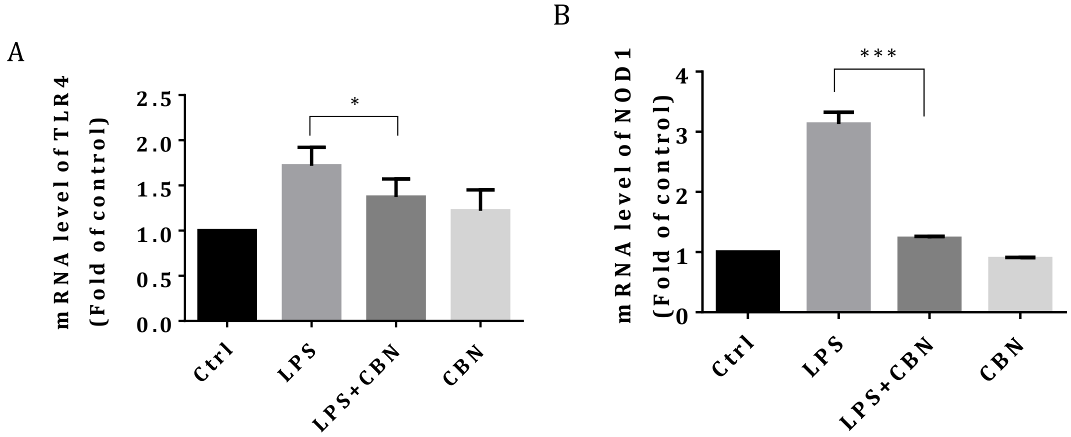

Fig 1. Effects of CBN on regulating TLR4 and NOD1 genes expression. CBN inhibited LPS-stimulated TLR4 (P<0.05) and NOD1 (P<0.001) genes expression. Results shown are representative of three independent experiments with similar results. Data are expressed as means ± SD.

Results and Conclusion:

We confirmed the RNA expression of TLR4. LPS stimulation significantly increased the gene expression of TLR4 (Fig A P<0.05). But, after the effect of CBN, the reduce level of TLR4 was significantly smaller than that in NOD1, which were markedly reduced by CBN treatment (Fig B P<0.001). Therefore, we hold the

opinion that, CBN primarily exerts its anti-inflammatory effects on NOD1 signaling pathway, maybe TLR4 pathway is a secondary target.
