# Supplementary material for: Columbianadin Suppresses Lipopolysaccharide (LPS)-Induced Inflammation and Apoptosis through the NOD1 Pathway
Source: Molecules. 2019 Feb 2;24(3):549. doi: 10.3390/molecules24030549 (PMC6384818; doi:10.3390/molecules24030549)
Supplement: Supplementary file 1 [file molecules-24-00549-s001.zip › molecules-432611-supplementary-edited-2.pdf]

Table S1. The following primers were used.

homo-NOD1-F1	5’-CACTCAGAGCAAAGTCGTGG-3’
homo-NOD1-R1	5’-CAGCACGAACTTGGAGTCAC-3’
homo-RIP2-F1	5’-GTCCCTCTCACAGTCACGAA-3’
homo-RIP2-R1	5’-TGCTTGATACTGGCCCTTGA-3’
homo-NFKB-P65-F1	5’-GTGAACCGAAACTCTGGCAG-3’
homo-NFKB-P65-R1	5’-ATCAGCTTGCGAAAAGGAGC-3’
homo-GAPDH-F1	5’-CATGAGAAGTATGACAACAGCCT-3’
homo-GAPDH-R1	5’-AGTCCTTCCACGATACCAAAGT-3’
